# Chinese translation of strengths and difficulties questionnaire requires urgent review before field trials for validity and reliability

**DOI:** 10.1186/1753-2000-2-23

**Published:** 2008-08-15

**Authors:** Teck-Hock Toh, Sing-Jill Chow, Tzer-Hwu Ting, Jill Sewell

**Affiliations:** 1 Fellow, Centre for Community Child Health, The Royal Children's Hospital, Parkville, Victoria, 3052, Australia; 2 Fellow, Department of Endocrinology, The Royal Children's Hospital, Parkville, Victoria, 3052, Australia; 3 Consultant Paediatrician, Centre for Community Child Health, The Royal Children's Hospital, and Principal Fellow, Faculty of Medicine, Dentistry and Health Sciences, University of Melbourne, Parkville, Victoria, 3052, Australia

## Abstract

Strengths and Difficulties Questionnaire (SDQ) is a brief behavioural screening questionnaire for children and teenagers aged 3 to 16 years. It is available in 66 languages, and gaining more popularity world wide. Chinese translation of SDQ is available and has been used in clinical practice and research. We undertook the exercise to back-translate the current Chinese translation and it showed a number of differences compared to the original English SDQ. The differences and concerns include: (1) the flow and grammar of Chinese translation as well as wrongly written Chinese characters; (2) translated words that have deviated from the original meaning; (3) significant numbers of wording that are somewhat different from the original English version; (4) addition of auxiliary verbs that do not exist in original English version; and (5) the current Chinese SDQ is a general questionnaire for all age groups that does not observe the differences of wording that exist in the English versions.

An accurate translated Chinese version is important for researchers, clinicians and educators who work in the Chinese communities. There is an urgent need to review the translation of the Chinese SDQ version before more studies use it in the field.

## Full Text

The results of a study in China on the validity, reliability and normative scores of a Chinese translation of the Strengths and Difficulties Questionnaire (SDQ) were recently published in your online journal on 29 April 2008 by Du *et al *[1]. Findings on psychometric properties were mixed, especially in the areas of peer problems and self-rating by adolescents. Concern was also raised about the validity of the Chinese translation. This does not surprise us because we believe the answers lie in the Chinese translation of the SDQ.

The SDQ is a brief behavioural screening questionnaire for children and teenagers aged 3 to16 years [2]. It was first tested in the United Kingdom and copy-righted by Goodman in 1997 [3]. Several versions are available and each version may include one to three of the following: a) 25-item psychological attributes, b) 5-question impact supplement, and c) seven follow-up questions. It is available in 66 languages, which include three English versions for the USA, United Kingdom and Australia that differ slightly in the wording used and age specification [2].

The Centre for Clinical Trials and Epidemiological Research at the Chinese University of Hong Kong and Iris Tan Mink had contributed greatly in the translation, back-translation and validation of the Chinese version [2]. Currently the Chinese translation has three versions available for parent, teacher and student respectively and each version consists of the 25-item psychological attributes and impact supplement only. They were presumably translated from the United Kingdom's English version because the wording matches more closely than the other English versions [2]. It is available in traditional form of Chinese writing, commonly used in Hong Kong and Taiwan. Chinese communities in China and South East Asian countries use the simplified form of writing.

Kou J, Du Y and Xia L published an article in Chinese in 2005 which concluded that the Chinese SDQ can be used to assess children and adolescents in Shanghai. This was derived from a validity and reliability study involving parents of 2128 students, using the three versions of Chinese SDQ and a retest 6 weeks later involving 47 of these parents [4].

Despite reported findings by Du Y and Kou J [1,4], we feel strongly that the current Chinese translation of SDQ has a number of differences compared to the original English SDQ. It is challenging and unscientific to compare any finding as a result of using two questionnaires with different languages and meaning. We therefore question the conclusion by Du Y *et al *about the use of Chinese version of SDQ in China.

We recognize that translation of scientific and clinical materials is not an easy task. We believe much effort has been put forward in the first translation by people in the Chinese University of Hong Kong and Iris Tan Mink. Their contribution should be recognized and appreciated. However, the current Chinese translation of SDQ should be critically appraised and reviewed to provide a more accurate translated Chinese version of SDQ that is reliable for its users in the field.

An exercise was undertaken by two authors of this letter (Toh TH and Ting TH) to back-translate the current Chinese SDQ independently. Ting TH had no prior knowledge of the SDQ before the translation. Both back-translations were similar, and they are presented in Additional files 1, 2 and 3. The differences and concerns we found are as follow:

1. The flow and grammar of the current Chinese SDQ are not smooth, with wrongly written Chinese characters.

2. Some translated word has deviated from the original meaning.

3. Significant numbers of wording, which include the word "*True*", used as the answer of all the 25 items, are somewhat different from the original UK English version.

4. Auxiliary verb "*will*", "*can*" and "*very*" were added in many of the 25-item psychological attributes and the significance of adding these verbs is unclear.

5. The current Chinese SDQ is a general questionnaire for all age groups, and does not observe some differences of wording that exists in the English versions.

Examples and explanation of these major differences and concerns are included in Table 1.

**Table 1:** Summary of differences found between original english (UK) SDQ and Chinese translation

	Major Differences and Concerns	Affected Items/Questions & Examples	Implications/ Suggestions
1	Chinese grammar/flow and wrongly written Chinese characters	○ Items 2, 7, 12 & 23 in Parent/Teacher version ○ Items 2, 12, 14, 17 & 23 in Student version ○ Question 1 to 4 of the impact supplement in all three versions ○ Two wrongly written Chinese characters (Item 15 in Student version and Question 4 in impact supplement, Parent/Teacher versions)	Can be improved and rephrased to a more comprehensible language and more easily understood by a lay audience

2	Deviation of translated word	○ Items 4, 7, 9, 12 & 17 in Parent/Teacher version* ○ Items 4, 8, 9, 10, 11, 12, 20 & 25 in Student version* ○ Question 1, 3 & 4 of the impact supplement in all three versions* ○ Introductory paragraph of the Student version*	These words need to be reviewed and matching the original English version

3	Translated word that is "somewhat different"	○ The answers to the 25 items, "*true*"^†^ ○ Items 3, 5, 6, 8, 13, 24 & 25 in Parent/Teacher version^‡^ ○ Items 6, 18 & 23 in Student version^‡^ ○ Question 1 of the impact supplement in all three versions^‡^	These words require further consideration and the significance of the differences is unclear.

4	Addition of auxiliary verbs ("*will", "can" *and *"very"*)	○ Items 16, 21 & 22 in Parent version ○ Items 16 & 21 in Teacher version ○ Items 16, 17, 21, 22 & 24 in Student version	The significance of these verbs is unclear, ideally they should be removed

5	Age-unspecific versions	The English versions are divided into different age groups, with some differences in wording. E.g., "*often argumentative with adults*" in the 3–4 years old group is represented by "*often lies or cheats*" in the 4–16 years old group. Current Chinese SDQ does not observe these differences because one version is used for all age groups. In this example, the item concerned was translated as "*often lies or cheats*" only.	

* For examples, instead of "*fights*", it was translated as "*quarrel*" and "*argue*"; instead of "upset", "*unwell*" and "*sad*" were used. "*I have one good friend or more*", was translated as "*I have one or a few good friends*"; and "*do the difficulties upset or distress your child?*" became "*are these difficulties perplexing/puzzling/disturbing you?*"

^† ^"*Not True*", "*Somewhat True*" and "*Certainly True*" – the answers to the 25 items, "*true*" was translated as "*tallying/accord or keeping with*"

^‡ ^For examples "*often seems worried*" was translated as "*often exhibit/display sign of anxiety*"; "*tearful*" was translated as "*crying*".

An online search on 12 June 2008, involving PsycINFO 1806, Ovid MEDLINE(R) 1996, CINAHL 1982 and EMBASE 1996 using "SDQ or Strengths and Difficulties Questionnaire" and "Chinese or Mandarin or China or Taiwan or Hong Kong" as key words have shown numerous publications quoting the use of SDQ Chinese translations in China and Hong Kong. It was used as a measurement tool for interventional trials [5,6] and descriptive epidemiological studies [7,8]. Clinicians have also used it as a screening tool to prioritise psychiatry services [9] and to compare findings on psychometric properties of parent ratings on the Chinese version of the Swanson, Nolan, and Pelham IV scale [10].

## Conclusion

It is obvious that SDQ will gain increasing popularity world wide, and an accurate translated Chinese version is important for researchers, clinicians and educationists who work in the Chinese population. There is an urgent need to review the translation of the Chinese SDQ version before more studies use it in the field. A more complete set of Chinese SDQ versions in both traditional and simplified Chinese forms of writing should be made available on the SDQ website.

## Competing interests

The authors declare that they have no competing interests.

## Authors' contributions

Back-translation of the Chinese SDQ was performed by Toh TH and Ting TH. In addition all authors have contributed towards the writing and approval of this letter.

## Availability & requirements





## Supplementary Material

Additional file 1 Appendix A. Click here for file

Additional file 2 Appendix B. Click here for file

Additional file 3 Appendix C. Click here for file
