# Supplementary material for: Chinese translation of strengths and difficulties questionnaire requires urgent review before field trials for validity and reliability
Source: Child Adolesc Psychiatry Ment Health. 2008 Aug 15;2:23. doi: 10.1186/1753-2000-2-23 (PMC2533285; doi:10.1186/1753-2000-2-23)
Supplement: Additional file 1 — Appendix A. [file 1753-2000-2-23-S1.doc]

**Appendix A:**

**Back-translation of SDQ Chinese version**

**(Comparing to UK English Version – T4-16 (c4))**

Strengths and Difficulties (Parental Version)

With regards to the following questions, please mark a cross in the relevant box, to indicate if your child _______________ (child’s name) fulfils the situation - as “not tallying/accord or keeping with”, “somewhat tally/accord or keeping with”, or “completely tally/accord or keeping with ”. Please answer the question based on your child’s last 6 months behaviour. Please make sure all questions are answered, even if you are not sure or unclear of particular questions.

Child’s name:

Date of birth:

Male / Female

- not tallying/accord or keeping with
- somewhat tally/accord or keeping with
- completely tally/accord or keeping with

| Understanding/Considerate of other’s feeling  Not settling / still (move a lot, restless), over active (hyperactive), can not be quiet/calm for long (Chinese can be improved)  Often complaint of headache, stomach ache or feeling not well  Very willing to share object/things with other children (sweets, toys, pencil, etc.)  Often lose temper (gets angry) or throw temper tantrum / make a fuss |
| --- |
| Quite isolated, more frequently/often play alone (or by oneself)  Generally speaking / in general, more obedient, usually willing to do what the adults request to do (follows an adult’s instruction) (Chinese can be improved)  Have a lot of worries, often exhibit/display (sign of) anxiety/anxious  If people are hurt/injured, not well / unwell or sick, is very keen/glad to provide help  Often fidget or move restless/move about/dashing around (associated with nervousness) |
| Has at least a good friend  Often quarrel with other children or bullies other children (Chinese can be improved)  Often unhappy, depressed (sad) or crying  Generally speaking / in general, liked by other children  Lose concentration easily, concentration is poor (unable to concentrate) |
| In new condition/situation/circumstances, will/can get nervous/anxious or stick/clingy to adult, lose confidence easily  Kind/friendly to young children  Often lies or cheat  Teased or bullied by other children  Often offer to help others (parents, teacher or other children) |
| Will/can think clearly before acting / doing things  Will/can steal from home, school or other places  Get alone with adults more harmoniously than with children (Chinese can be improved)  Feels scared / afraid of many things, easily frightened  Can do till the end when doing thing, has long concentration span |

Do you have other opinion/comment ________________________________

Please turn over

Do you think your child has the following difficulties? Difficulties in the areas of emotion, concentration, behaviour, or getting alone (interact) with other people (Chinese can be improved)

- No
- Yes - has a little difficulty (Chinese can be improved)
- Yes – has difficulties
- Yes – has a lot of difficulties

If your answer is “yes”, please answer the following questions regarding these difficulties

How long have these difficulties appeared/existed? (Chinese can be improved)

- Less than a month
- 1 to 5 months
- 6 to 11 months
- More than 1 year and beyond (Chinese can be improved)

Are these difficulties perplexing/puzzling/disturbing your child?

- No
- A little / slightly
- Quiet a lot
- Very much

Are these difficulties causing/resulting in any disturbances / interference in your child’s day to day life in the following areas? (Chinese can be improved – probably a typo also)

- No
- Slightly / a little
- Quiet a lot
- Very much
- Family life
- Interaction with friends (Chinese can be improved)
- Classroom learning
- Extra-curricular activities

Have these difficulties added burden to yourself or your family?

- No
- Slightly / a little
- Quiet a lot
- Very much

Signature: ………………………. Date: ……………………….

Father/mother/other (please specify)

Thanks very much for your help!

-----------------------------------------------------------------------------------------------------------

**Authors’ Note:**

The back-translation was completed by TH TING (who has no previous knowledge of SDQ) and TH TOH independently. Both translations are very similar (with minor words difference – in such case, the differences are presented together). The coloured remarks are interpreted as follow:

Yellow = minor difference, can accept Pink = moderate difference, ? accept

Red = major difference, not acceptable Brown = auxiliary verbs

References:

- Xin Hua Ci Dian, Beijing Shang Wu Publisher (Chinese Dictionary), 2001
- A Modern Chinese English Dictionary, Beijing Foreign Language Teaching and Research Press, China), November 1995
