# Supplementary material for: Chinese translation of strengths and difficulties questionnaire requires urgent review before field trials for validity and reliability
Source: Child Adolesc Psychiatry Ment Health. 2008 Aug 15;2:23. doi: 10.1186/1753-2000-2-23 (PMC2533285; doi:10.1186/1753-2000-2-23)
Supplement: Additional file 3 — Appendix C. [file 1753-2000-2-23-S3.doc]

**Appendix C:**

**Back-translation of SDQ Chinese version**

**(Comparing to UK English Version – S11-16 (c8))**

Strengths and Difficulties (Student Version)

According to your experience and facts within the last 6 months, please answer the following question, please pick an answer that you feel is appropriate from the options listed on the right of the questions – “not tallying/accord or keeping with”, “somewhat tally/accord or keeping with”, or “completely tally/accord or keeping with ”. Please do not leave any question unanswered, even if you are not sure of particular questions.

Your name:

Date of birth:

Male / Female

- not tallying/accord or keeping with
- somewhat tally/accord or keeping with
- completely tally/accord or keeping with

| I try to be nice/friendly to people, I concern/care about other’s (people’s) feeling  I can not stay still, can not be quiet (calm) for long time (Chinese can be improved)  I often get (have) headache, stomach ache or feeling unwell  I often share with people (food, toys, pen, etc.)  I feel very angry and often lose my temper |
| --- |
| I often stay alone. I usually play alone (or by myself)  I usually do according to the request (I often do what I am told to)  I am often worried, laden with anxiety  I am willing to help someone who is injured, sad (feel sorry) or unwell  I often fidget or feel inpatient |
| I have one or a few good friends  I often have argue (dispute/disagree) with others. I can make other people do what I want them to (Chinese can be improved)  I often feel unhappy, depressed (having a heavy heart) or tearing  Generally speaking / in general, other people who are close to my age like me (Chinese can be improved)  I lose concentration/(am) distracted easily, I feel difficult to concentrate (A Chinese typo) |
| I will/can feel nervous when I am in a new environment/circumstance/surrounding. I lose confidence very easily  I will/can treat kindly children younger than I am (Chinese can be improved)  I am often criticized/accused (but in lesser tone) of lying or being dishonest  Other children or youth often tease (pick on) or bully me  I often offer to help other people (parents, teacher, classmates) |
| I will/can think clearly before acting / doing things  I will/can take things which are not mine from home, school or other places  I get along (interact) more harmoniously (better) with adult rather than with people of my generation (peers) (Chinese can be improved)  There are many fears in my heart (I have many fears). I am very easily frightened  I can always finish my work well. My concentration is good. |

Do you have other opinion/comment ________________________________

Please turn over

In summary (not a commonly use lay term), do you think you have difficulties in the following areas? Areas of emotion, concentration, behaviour, or getting alone harmoniously with other people (Chinese can be improved)

- No
- Yes - has a little difficulty (Chinese can be improved)
- Yes – has difficulties
- Yes – has a lot of difficulties

If your answer to the above question is “yes”, please answer the following questions regarding these difficulties

How long have these difficulties appeared/existed? (Chinese can be improved)

- Less than a month
- 1 to 5 months
- 6 to 11 months
- More than 1 year and beyond (Chinese can be improved)

Are these difficulties perplexing/puzzling/disturbing you?

- No
- A little / slightly
- Quiet a lot
- Very much

Are these difficulties causing/resulting in any disturbances / interference in your day to day life in the following aspects? (Chinese can be improved)

- No
- Slightly / a little
- Quiet a lot
- Very much
- Family life
- Interaction with friends (Chinese can be improved)
- Classroom learning
- Extra-curricular leisure activities

Do your difficulties become a burden to others around you (family members, friends, teacher/senior etc)?

- No
- Slightly / a little
- Quiet a lot
- Very much

Signature: ………………………. Date: ……………………….

Thanks very much for your help!

-----------------------------------------------------------------------------------------------------------

**Authors’ Note:**

The back-translation was completed by TH TING (who has no previous knowledge of SDQ) and TH TOH independently. Both translations are very similar (with minor words difference – in such case, the differences are presented together). The coloured remarks are interpreted as follow:

Yellow = minor difference, can accept Pink = moderate difference, ? accept

Red = major difference, not acceptable Brown = auxiliary verbs

References:

- Xin Hua Ci Dian, Beijing Shang Wu Publisher (Chinese Dictionary), 2001
- A Modern Chinese English Dictionary, Beijing Foreign Language Teaching and Research Press, China), November 1995
